# Supplementary material for: FastqPuri: high-performance preprocessing of RNA-seq data
Source: BMC Bioinformatics. 2019 May 3;20:226. doi: 10.1186/s12859-019-2799-0 (PMC6500068; doi:10.1186/s12859-019-2799-0)
Supplement: Supplementary file 2 — Archive of FastqPuri. Archive containing all files needed to install and run FastqPuri v1.0.6. Date stamp March 22, 2019. (GZ 47,819 kb) [file 12859_2019_2799_MOESM2_ESM.gz › FastqPuri-1.0.6/html/md_README_makeBloom.html]

My Project: makeBloom user manual


My Project

makeBloom user manual

Reads a `fasta` file, creates a bloom filter with a predefined:

- size (bits).
- number of hash functions.
- false positive rate. and saves it to a file.

## Running the program

Usage `C` executable (in folder `bin`):

Usage: ./makeBloom --fasta <FASTA\_INPUT> --output <FILTERFILE> --kmersize [KMERSIZE]

(--fal\_pos\_rate [p] | --hashNum [HASHNUM] | --bfsizeBits [SIZEBITS])

Options:

-v, --version Prints package version.

-h, --help Prints help dialog.

-f, --fasta Fasta input file. Mandatory option.

-o, --output Output file, with NO extension. Mandatory option.

-k, --kmersize kmer size, number or elements. Optional(default = 25)

-p, --fal\_pos\_rate false positive rate. Optional (default = 0.05)

-g, --hashNum number of hash functions used. Optional (default

value computed from the false positive rate).

-m, --bfsizeBits size of the filter in bits. It will be forced to be

a multiple of 8. Optional (default value computed

from the false positive rate).

NOTE: the options -p, -n, -m are mutually exclusive. The program

will give an error if more than one of them are passed as input.

It is recommended to pass the false positive rate and let the

program compute the other variables (excepting singular situations)

If none of them are passed, the false positive rate will default

to 0.05 and the other variables will be computed respecting this

requirement. See documentation and supplementary material for

more details.

## Output description

Two files are created as output:

- `<FILTERFILE>.bf`: contains the filter (binary file).
- `<FILTERFILE>.txt`: contains the following information:
  - `kmersize`: length of the kmers inserted in the filter,
  - `bfsizeBits`: size of Bloom filter in bits,
  - `hashNum`: number of hash functions used in the filter,
  - `falsePosRate:` false positive rate,
  - `nelem`: number of elemens (kmers in the sequece) contained in the filter.

For further details, read the `Doxygen` documentation of the files `bloom.c`, `init_makeBloom.c`, `makeBloom.c`

## Example

In the folder `../examples/bloomROC/` an example script can be run to test the bloom filter performance.   
- The fastq files:

- `human_reads.fq.gz`
- `EColi_reads.fq.gz` are analyzed. They contain 10e5 reads each generated with `dgwsim`.

STEP1: Create bloom filters for EColi genome with FPR: `[0.005 0.0075 0.01 0.02]`

- STEP2: Run trimFilter on both data looking for contaminations from EColi using all filters generated, with `kmersize = 25` and scores ranging from `0.05` to `0.2` by `0.01` intervals. We obtain false/true positive/negative rates:
  - FPR: % contaminations detected in human\_reads.fq.gz
  - TNR (specificity): % good reads detected in `human_reads.fq.gz`
  - FNR: % good reads detected in `EColi_reads.fq.gz`
  - TPR (sensitivity): % contaminations detected in `EColi_reads.fq.gz`
- STEP3: Create ROC curves for all filters (sensitivity vs FPR).   
  The results (`*csv`, `*pdf`) can be compared with `example*pdf`, and `example*csv` in the folder (see example for `-p = 0.0075` below).

## Details on bloom filters

**NOTE**: For further details, read the `Doxygen` documentation of the files `bloom.c`, `init_makeBloom.c`, `makeBloom.c` and the supplementary material.

A bloom filter is a probabilistic data structure used to test if an element is a member of a set. False positive matches are possible but false negative are not. For a given set of `n` elements, we proceed as follows:

- decide on the number `g` of hash functions we will use for the construction of the filter and the length of the filter `m` (number of bits). This choice will be made based on the false positive rate we want to achieve (see Parameters)
- we create an empty bloom filter, `B`, i.e. an array of `m` bits set to `0`. For every element in the set, **s_α_ ∈ S**, compute the `g` hash functions, **H_i_ (s_α_) ∀ i ∈ {1,...,g}** and set the corresponding bits to `1` in the filter, i.e., **B[H_i_ (s_α_) mod m] = 1 ∀ i ∈ {1,...,g}** and `0` otherwise.

Then, if we want to check whether an element **s_β_** is in the set **S**, we compute **H_i_ (s_β_) ∀ i ∈ {1,...,g}** and check whether all coresponding positions in the filter are set to `1`, in which case we can say that **s_β_** might be in the set. Otherwise it is definitely not in the set.

### Parameters.

We choose the parameters so that the desired false positive rate is achieved. Alternatively, we can pass the filter size, and then the number of hash functions to be used is tuned so that the false positive rate is minimized.

We assume the hash functions select all positions with the same probability. The probability that an bit in the filter `B` is not set to `1` after inserting an element using `g` hash functions is:

**(1 - ^1^⁄_m_)^g^**

where `m` is the number of bits of the filter. If we insert `n` elements, the probability that an element is still 0 is:

**p_0_ = (1 - ^1^⁄_m_)^gn^**

The probability that a bit is `1` is then,

**p_1_ = 1 - (1 - ^1^⁄_m_)^gn^**

Now, let's compute the false positive rate, i.e., that probability that an element that is not in the set is classified as belonging to it. This is the probability that all positions computed from the hash functions being `1` is,

**p(g, n, m) = (1 - (1 - ^1^⁄_m_)^gn^)^g^ = (1 - e ^- gn⁄_m_^ )^g^**

For a given `n` and `m` the value of `g` that minimizes `p` is,

**^dp(g, n, m)^⁄_dg_ = 0 ⇒ g = ^m^⁄_n_ log(2)**

The required number of bits `m` for the desired positive rate given `n` number of elements and assuming the optimal number of hash functions being used is,

**m = - ^n log (p)^⁄_log^2^(2)_.**

### Creating a bloom filter from a `fasta` file

Given a `fasta` file, the elements to be inserted in the bloom filter are all possible `k`-mers contained in the `fasta` file. The length `k` of the `k`-mers can be given by the user as an input parameter and is chosen to be `25` by default. All `k`-mers containing nucleotides different from `{A,C,G,T}` will not be consiedered and they are encoded such that every nucleotide takes only 2-bits memory. Wee look into the reverse complement and insert only the one that is lexycographically smaller.

Once the `k`-mer has been processed, the hash functions are computed an the positions of the output values are set to `1` in the filter.

### Checking ir a read in a `fastq` file is in the filter

To check whether a `fastq` read of length `L` is in the filter, we proceed as follows:

1. Start with a score `score = 0`.
2. Construct and process all `k`-mers of length `k` in the read, `(L - k + 1)` (note that `L`≥`k`)
3. For each `k`-mer, compute the `g` hash functions and check whether all bits in the corresponding positions in the filter are set to `1`. If so, add \*\*^1^⁄_(L - k + 1)_\*\* to the score.

If the score is above the user predefined threshold (`-s`), the read is classified as belonging to the set, and not otherwise.

### Memory usage, sensitivity and specificity

The **memory usage** will be determined by `m`, the size of the filter. The optimal number of bits per element is

**^m^⁄_n_ = -  ^log (p)^⁄ _log^2^(2)_.**

In the figures below, we can see both, the optimal number of bits per element and the optimal number of hash functions as a function of the false positive rate.

As an example, let's assume we want to look for contaminations in a genome `~3GB` and want to keep the false positive rate by `2%`. Then, we will need a filter of `~3.05GB`.

**Sensitivity** (true positive rate, TP/(TP + FN)) can be increased by decreasing the `k`-mer size (`-k`) and the score threshold. False negatives only occur in the presence of mismatches due to variants, or errors in the base calling procedure, since the filter itself does not allow for false negatives.

To increase **specificity** (true negative rate, TN/(TN + FP)), you can increase the score threshold (`-s`) or, obviously reduce the positive rate, (`-p`).

## Contributors

Paula Pérez Rubio

## License

GPL v3 (see LICENSE.txt)


---

Generated by  

 1.8.14
